# Diethyl 4-[5-(4-chloro­phen­yl)-1*H*-pyrazol-4-yl]-2,6-dimethyl-1,4-dihydro­pyridine-3,5-dicarboxyl­ate

**DOI:** 10.1107/S160053681201344X

**Published:** 2012-04-04

**Authors:** Hoong-Kun Fun, Wan-Sin Loh, A. M. Vijesh, Arun M. Isloor, Shridhar Malladi

**Affiliations:** a X-ray Crystallography Unit, School of Physics, Universiti Sains Malaysia, 11800 USM, Penang, Malaysia; b Department of Chemistry, National Institute of Technology-Karnataka, Surathkal, Mangalore 575 025, India

## Abstract

In the title compound, C_22_H_24_ClN_3_O_4_, intra­molecular C—H⋯O and C—H⋯N hydrogen bonds form *S*(9) and *S*(7) ring motifs, respectively. The 1,4-dihydro­pyridine ring adopts a flattened boat conformation. The benzene ring makes a dihedral angle of 33.36 (6)° with the pyrazole ring. In the crystal, pairs of N—H⋯N hydrogen bonds link the mol­ecules into inversion dimers. The dimers are stacked in column along the *a* axis through N—H⋯O and C—H⋯N hydrogen bonds. The crystal packing also features C—H⋯π inter­actions involving the pyrazole ring.

## Related literature

For background to and applications of 1,4-dihydro­pyridines, see: Janis & Triggle (1983 ▶); Boecker & Guengerich (1986 ▶); Gordeev *et al.* (1996 ▶); Buhler & Kiowski (1987 ▶); Vo *et al.* (1995 ▶). For hydrogen-bond motifs, see: Bernstein *et al.* (1995 ▶). For ring conformations, see: Cremer & Pople (1975 ▶). For a related structure, see: Fun *et al.* (2012 ▶). For the stability of the temperature controller used in the data collection, see: Cosier & Glazer (1986 ▶).
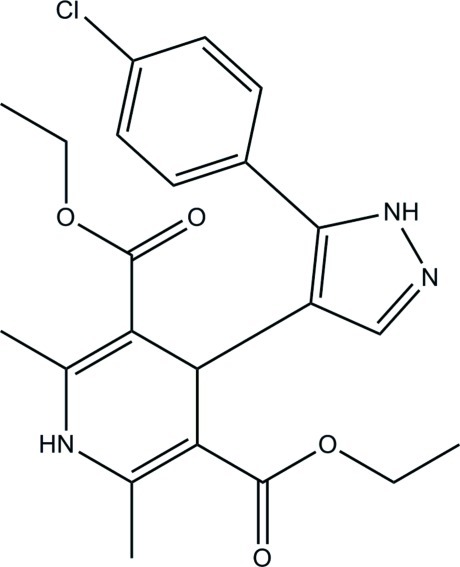


## Experimental

### 

#### Crystal data

C_22_H_24_ClN_3_O_4_
*M* *_r_* = 429.89
Triclinic, 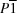

*a* = 8.5210 (5) Å
*b* = 10.7809 (6) Å
*c* = 11.2707 (7) Å
α = 90.411 (1)°
β = 97.205 (1)°
γ = 94.210 (1)°
*V* = 1024.28 (10) Å^3^
*Z* = 2
Mo *K*α radiation
μ = 0.22 mm^−1^
*T* = 100 K
0.38 × 0.18 × 0.17 mm

#### Data collection

Bruker SMART APEXII DUO CCD area-detector diffractometer
Absorption correction: multi-scan (*SADABS*; Bruker, 2009 ▶) *T*_min_ = 0.921, *T*_max_ = 0.964
17114 measured reflections
5885 independent reflections
5038 reflections with *I* > 2σ(*I*)
*R*_int_ = 0.027

#### Refinement

*R*[*F* ^2^ > 2σ(*F* ^2^)] = 0.038
*wR*(*F* ^2^) = 0.107
*S* = 1.04
5885 reflections
283 parameters
H atoms treated by a mixture of independent and constrained refinement
Δρ_max_ = 0.44 e Å^−3^
Δρ_min_ = −0.29 e Å^−3^

### 

Data collection: *APEX2* (Bruker, 2009 ▶); cell refinement: *SAINT* (Bruker, 2009 ▶); data reduction: *SAINT*; program(s) used to solve structure: *SHELXTL* (Sheldrick, 2008 ▶); program(s) used to refine structure: *SHELXTL*; molecular graphics: *SHELXTL*; software used to prepare material for publication: *SHELXTL* and *PLATON* (Spek, 2009 ▶).

## Supplementary Material

Crystal structure: contains datablock(s) global, I. DOI: 10.1107/S160053681201344X/is5103sup1.cif

Structure factors: contains datablock(s) I. DOI: 10.1107/S160053681201344X/is5103Isup2.hkl

Supplementary material file. DOI: 10.1107/S160053681201344X/is5103Isup3.cml

Additional supplementary materials:  crystallographic information; 3D view; checkCIF report

## Figures and Tables

**Table 1:** Hydrogen-bond geometry (Å, °) *Cg*1 is the centroid of the N1/N2/C7–C9 ring.

*D*—H⋯*A*	*D*—H	H⋯*A*	*D*⋯*A*	*D*—H⋯*A*
N1—H1*N*1⋯O4^i^	0.857 (17)	2.078 (17)	2.9291 (14)	172.2 (17)
N3—H1*N*3⋯N2^ii^	0.908 (19)	2.184 (19)	3.0427 (14)	157.5 (15)
C5—H5*A*⋯O1	0.93	2.27	3.1988 (16)	177
C8—H8*A*⋯N3	0.93	2.61	3.2546 (15)	127
C22—H22*B*⋯N2^iii^	0.96	2.50	3.3741 (16)	151
C19—H19*B*⋯*Cg*1^iv^	0.96	2.79	3.5562 (14)	137

Symmetry codes: (i) 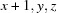; (ii) 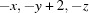; (iii) 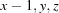; (iv) 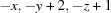.

**Fig. 1.**
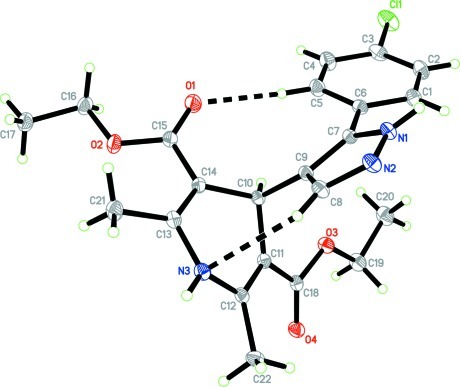
The molecular structure of the title compound, showing 50% probability displacement ellipsoids. Dashed lines indicate the intramolecular hydrogen bonds.

**Fig. 2.**
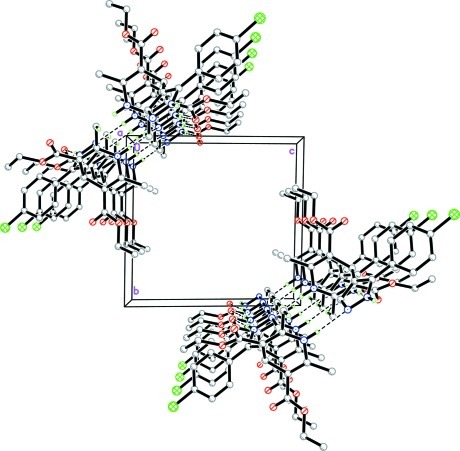
A part of crystal packing diagram of the title compound, viewed along the a axis. H atoms not involved in the intermolecular interactions (dashed lines) have been omitted for clarity.

## supplementary crystallographic information

### Comment

In recent years, considerable attention has been paid to the synthesis of 1,4-dihydropyridines owing to their significant biological activity. 1,4-Dihydropyridine-containing drugs (1,4-DHPs), such as nifedipine, nicardipine, amlodipine, felodipine and others have been found to be useful as calcium channel blockers (Janis & Triggle, 1983; Boecker & Guengerich, 1986; Gordeev *et al.*, 1996) and are used most frequently as cardiovascular agents for the treatment of hypertension (Buhler & Kiowski, 1987). A number of DHP derivatives are employed as potential drug candidates for the treatment of congestive heart failure (Vo *et al.*, 1995). Prompted by the diverse activities of 1,4-dihydropyridines, we have synthesized the title compound to study its crystal structure.

In the title compound (Fig. 1), intramolecular C5—H5A···O1 and C8—H8A···N3 hydrogen bonds form *S*(9) and *S*(7) ring motifs (Bernstein *et al.*, 1995), respectively. The 1,4-dihydropyridine ring (C10–C12/N3/C13/C14) adopts a flattened boat conformation (Cremer & Pople, 1975) with the puckering parameters, Q = 0.4162 (11) Å; Θ = 74.64 (16)°; φ = 176.34 (17)°. The benzene ring (C1–C6) forms a dihedral angle of 33.36 (6)° with the pyrazole ring (N1/N2/C7–C9). The bond lengths and angles are within the normal ranges and are comparable with the related structure (Fun *et al.*, 2012).

In the crystal packing (Fig. 2), intermolecular N1—H1N1···O4, N3—H1N3···N2 and C22—H22B···N2 hydrogen bonds (Table 1) link the molecules into a column along the *a* axis. The crystal packing is further stabilized by C—H···π interactions (Table 1) involving the pyrazole ring.

### Experimental

3-(4-Chlorophenyl)-1*H*-pyrazole-4-carbaldehyde (0.2 g, 1.1 mmol), ethylacetoacetate (0.3 g, 2.3 mmol) and ammonium acetate (0.09 g, 1.2 mmol) in ethanol (20 ml) were refluxed for 8 h in an oil bath. After the completion of the reaction, the reaction mixture was concentrated and poured into crushed ice. The precipitated product was filtered and washed with water. The resulting solid was recrystallized from hot ethanol (0.33 g, 67%). *M.p.*: 459–461 K.

### Refinement

N-bound H atoms were located in a difference Fourier map and were refined freely [N—H = 0.80 (3) to 0.87 (3) Å]. The remaining H atoms were positioned geometrically (C—H = 0.93 to 0.97 Å) and refined with a riding model with *U*_iso_(H) = 1.2 or 1.5*U*_eq_(C). A rotating group model was applied to the methyl groups. In the final refinement, twelve outliners were omitted, -2 -3 2, 1 -1 1, 4 -2 6, 2 -1 3, -3 -4 5, -3 -6 4, -3 -5 4, 4 -2 5, -2 -2 3, -1 -5 1, 0 4 1 and -2 -6 3.

### Crystal data

**Table:**

C_22_H_24_ClN_3_O_4_	*Z* = 2
*M**_r_* = 429.89	*F*(000) = 452
Triclinic, *P*1	*D*_x_ = 1.394 Mg m^−^^3^
Hall symbol: -P 1	Mo *K*α radiation, λ = 0.71073 Å
*a* = 8.5210 (5) Å	Cell parameters from 7118 reflections
*b* = 10.7809 (6) Å	θ = 2.4–32.6°
*c* = 11.2707 (7) Å	µ = 0.22 mm^−^^1^
α = 90.411 (1)°	*T* = 100 K
β = 97.205 (1)°	Block, colourless
γ = 94.210 (1)°	0.38 × 0.18 × 0.17 mm
*V* = 1024.28 (10) Å^3^	

### Data collection

**Table:**

Bruker SMART APEXII DUO CCD area-detector diffractometer	5885 independent reflections
Radiation source: fine-focus sealed tube	5038 reflections with *I* > 2σ(*I*)
Graphite monochromator	*R*_int_ = 0.027
φ and ω scans	θ_max_ = 30.0°, θ_min_ = 1.8°
Absorption correction: multi-scan (*SADABS*; Bruker, 2009)	*h* = −11→11
*T*_min_ = 0.921, *T*_max_ = 0.964	*k* = −15→15
17114 measured reflections	*l* = −15→15

### Refinement

**Table:**

Refinement on *F*^2^	Primary atom site location: structure-invariant direct methods
Least-squares matrix: full	Secondary atom site location: difference Fourier map
*R*[*F*^2^ > 2σ(*F*^2^)] = 0.038	Hydrogen site location: inferred from neighbouring sites
*wR*(*F*^2^) = 0.107	H atoms treated by a mixture of independent and constrained refinement
*S* = 1.04	*w* = 1/[σ^2^(*F*_o_^2^) + (0.0519*P*)^2^ + 0.4397*P*] where *P* = (*F*_o_^2^ + 2*F*_c_^2^)/3
5885 reflections	(Δ/σ)_max_ = 0.001
283 parameters	Δρ_max_ = 0.44 e Å^−^^3^
0 restraints	Δρ_min_ = −0.29 e Å^−^^3^

### Special details

**Table:**

Experimental. The crystal was placed in the cold stream of an Oxford Cryosystems Cobra open-flow nitrogen cryostat (Cosier & Glazer, 1986) operating at 100.0 (1) K.
Geometry. All e.s.d.'s (except the e.s.d. in the dihedral angle between two l.s. planes) are estimated using the full covariance matrix. The cell e.s.d.'s are taken into account individually in the estimation of e.s.d.'s in distances, angles and torsion angles; correlations between e.s.d.'s in cell parameters are only used when they are defined by crystal symmetry. An approximate (isotropic) treatment of cell e.s.d.'s is used for estimating e.s.d.'s involving l.s. planes.
Refinement. Refinement of *F*^2^ against ALL reflections. The weighted *R*-factor *wR* and goodness of fit *S* are based on *F*^2^, conventional *R*-factors *R* are based on *F*, with *F* set to zero for negative *F*^2^. The threshold expression of *F*^2^ > σ(*F*^2^) is used only for calculating *R*-factors(gt) *etc*. and is not relevant to the choice of reflections for refinement. *R*-factors based on *F*^2^ are statistically about twice as large as those based on *F*, and *R*- factors based on ALL data will be even larger.

### Fractional atomic coordinates and isotropic or equivalent isotropic displacement parameters (Å^2^)

**Table:**

	*x*	*y*	*z*	*U*_iso_*/*U*_eq_	
Cl1	0.40990 (4)	0.46308 (3)	0.70565 (3)	0.02708 (9)	
O1	−0.04200 (12)	0.49719 (9)	0.17848 (9)	0.0248 (2)	
O2	−0.23718 (10)	0.49196 (8)	0.02494 (8)	0.01812 (18)	
O3	−0.12624 (10)	0.84126 (8)	0.44891 (7)	0.01690 (17)	
O4	−0.34579 (10)	0.94038 (8)	0.39174 (8)	0.01770 (17)	
N1	0.32527 (12)	0.89803 (9)	0.27948 (9)	0.01440 (19)	
N2	0.26859 (12)	0.96583 (10)	0.18490 (9)	0.01621 (19)	
N3	−0.24741 (11)	0.87179 (9)	0.03539 (9)	0.01423 (19)	
C1	0.36646 (14)	0.76773 (12)	0.50778 (10)	0.0169 (2)	
H1A	0.4078	0.8501	0.5120	0.020*	
C2	0.41253 (14)	0.68690 (12)	0.59919 (11)	0.0197 (2)	
H2A	0.4850	0.7149	0.6642	0.024*	
C3	0.34984 (14)	0.56477 (12)	0.59271 (11)	0.0192 (2)	
C4	0.24000 (15)	0.52106 (12)	0.49769 (12)	0.0213 (2)	
H4A	0.1967	0.4392	0.4955	0.026*	
C5	0.19539 (14)	0.60170 (12)	0.40547 (11)	0.0184 (2)	
H5A	0.1232	0.5729	0.3406	0.022*	
C6	0.25797 (13)	0.72537 (11)	0.40942 (10)	0.0139 (2)	
C7	0.21629 (13)	0.80907 (10)	0.31024 (10)	0.0126 (2)	
C8	0.11965 (13)	0.91862 (11)	0.15670 (10)	0.0156 (2)	
H8A	0.0501	0.9477	0.0947	0.019*	
C9	0.07844 (13)	0.81976 (10)	0.23091 (10)	0.0127 (2)	
C10	−0.08660 (12)	0.75561 (10)	0.22759 (9)	0.0122 (2)	
H10A	−0.0865	0.6963	0.2931	0.015*	
C11	−0.20389 (13)	0.85225 (10)	0.24478 (10)	0.0127 (2)	
C12	−0.26584 (12)	0.91545 (10)	0.14796 (10)	0.0129 (2)	
C13	−0.21330 (13)	0.74984 (11)	0.01598 (10)	0.0144 (2)	
C14	−0.14542 (13)	0.68669 (10)	0.11005 (10)	0.0132 (2)	
C15	−0.13184 (13)	0.55150 (11)	0.10873 (10)	0.0156 (2)	
C16	−0.24515 (15)	0.35748 (11)	0.02645 (11)	0.0192 (2)	
H16A	−0.2669	0.3273	0.1041	0.023*	
H16B	−0.1459	0.3273	0.0090	0.023*	
C17	−0.37844 (15)	0.31492 (12)	−0.06902 (12)	0.0209 (2)	
H17A	−0.3932	0.2258	−0.0697	0.031*	
H17B	−0.3528	0.3425	−0.1456	0.031*	
H17C	−0.4743	0.3494	−0.0526	0.031*	
C18	−0.23545 (13)	0.88366 (10)	0.36557 (10)	0.0138 (2)	
C19	−0.14246 (15)	0.86913 (13)	0.57282 (10)	0.0204 (2)	
H19A	−0.2427	0.8322	0.5932	0.024*	
H19B	−0.1386	0.9583	0.5862	0.024*	
C20	−0.00580 (16)	0.81475 (14)	0.64767 (11)	0.0240 (3)	
H20A	−0.0082	0.8352	0.7304	0.036*	
H20B	0.0924	0.8484	0.6233	0.036*	
H20C	−0.0145	0.7260	0.6370	0.036*	
C21	−0.25953 (15)	0.70438 (12)	−0.11096 (10)	0.0185 (2)	
H21A	−0.1851	0.6476	−0.1315	0.028*	
H21B	−0.2592	0.7739	−0.1637	0.028*	
H21C	−0.3638	0.6626	−0.1187	0.028*	
C22	−0.34816 (14)	1.03355 (11)	0.14701 (11)	0.0170 (2)	
H22A	−0.3285	1.0722	0.2250	0.026*	
H22B	−0.4602	1.0152	0.1260	0.026*	
H22C	−0.3086	1.0889	0.0895	0.026*	
H1N1	0.424 (2)	0.9115 (16)	0.3055 (16)	0.024 (4)*	
H1N3	−0.283 (2)	0.9163 (17)	−0.0293 (17)	0.028 (4)*	

### Atomic displacement parameters (Å^2^)

**Table:**

	*U*^11^	*U*^22^	*U*^33^	*U*^12^	*U*^13^	*U*^23^
Cl1	0.02373 (16)	0.03297 (19)	0.02383 (16)	0.00309 (12)	−0.00125 (11)	0.01615 (13)
O1	0.0259 (5)	0.0178 (4)	0.0280 (5)	0.0062 (4)	−0.0099 (4)	−0.0029 (4)
O2	0.0228 (4)	0.0133 (4)	0.0165 (4)	−0.0005 (3)	−0.0031 (3)	−0.0010 (3)
O3	0.0169 (4)	0.0239 (4)	0.0101 (4)	0.0046 (3)	0.0008 (3)	−0.0005 (3)
O4	0.0135 (4)	0.0216 (4)	0.0182 (4)	0.0023 (3)	0.0025 (3)	−0.0019 (3)
N1	0.0114 (4)	0.0169 (5)	0.0144 (4)	0.0002 (3)	−0.0002 (3)	0.0025 (3)
N2	0.0139 (4)	0.0187 (5)	0.0157 (4)	0.0008 (4)	0.0009 (3)	0.0051 (4)
N3	0.0151 (4)	0.0149 (5)	0.0123 (4)	0.0010 (3)	0.0002 (3)	0.0033 (3)
C1	0.0162 (5)	0.0192 (5)	0.0146 (5)	0.0001 (4)	0.0001 (4)	0.0015 (4)
C2	0.0170 (5)	0.0265 (6)	0.0145 (5)	−0.0001 (4)	−0.0020 (4)	0.0033 (4)
C3	0.0170 (5)	0.0244 (6)	0.0164 (5)	0.0043 (4)	0.0013 (4)	0.0089 (4)
C4	0.0204 (6)	0.0190 (6)	0.0230 (6)	−0.0005 (4)	−0.0023 (4)	0.0058 (5)
C5	0.0182 (5)	0.0178 (5)	0.0179 (5)	0.0009 (4)	−0.0026 (4)	0.0023 (4)
C6	0.0129 (5)	0.0173 (5)	0.0117 (5)	0.0029 (4)	0.0014 (4)	0.0019 (4)
C7	0.0120 (5)	0.0138 (5)	0.0120 (5)	0.0012 (4)	0.0009 (4)	0.0006 (4)
C8	0.0132 (5)	0.0183 (5)	0.0148 (5)	0.0010 (4)	0.0002 (4)	0.0038 (4)
C9	0.0125 (5)	0.0138 (5)	0.0116 (5)	0.0016 (4)	0.0006 (4)	0.0006 (4)
C10	0.0119 (4)	0.0132 (5)	0.0112 (4)	0.0006 (4)	0.0004 (3)	0.0008 (4)
C11	0.0111 (4)	0.0138 (5)	0.0130 (5)	−0.0003 (4)	0.0011 (4)	0.0000 (4)
C12	0.0100 (4)	0.0137 (5)	0.0146 (5)	−0.0007 (4)	0.0007 (4)	0.0009 (4)
C13	0.0136 (5)	0.0159 (5)	0.0132 (5)	−0.0007 (4)	0.0010 (4)	0.0002 (4)
C14	0.0127 (5)	0.0139 (5)	0.0127 (5)	0.0000 (4)	0.0008 (4)	−0.0006 (4)
C15	0.0147 (5)	0.0165 (5)	0.0154 (5)	0.0007 (4)	0.0014 (4)	−0.0020 (4)
C16	0.0239 (6)	0.0130 (5)	0.0197 (5)	0.0011 (4)	−0.0017 (4)	−0.0010 (4)
C17	0.0238 (6)	0.0172 (6)	0.0205 (6)	0.0001 (4)	−0.0015 (4)	−0.0022 (4)
C18	0.0125 (5)	0.0144 (5)	0.0137 (5)	−0.0016 (4)	0.0005 (4)	0.0003 (4)
C19	0.0195 (5)	0.0311 (7)	0.0110 (5)	0.0035 (5)	0.0025 (4)	−0.0025 (4)
C20	0.0230 (6)	0.0344 (7)	0.0138 (5)	0.0035 (5)	−0.0013 (4)	−0.0006 (5)
C21	0.0226 (6)	0.0200 (6)	0.0121 (5)	0.0006 (4)	−0.0001 (4)	0.0009 (4)
C22	0.0161 (5)	0.0163 (5)	0.0187 (5)	0.0035 (4)	0.0009 (4)	0.0033 (4)

### Geometric parameters (Å, º)

**Table:**

Cl1—C3	1.7387 (12)	C9—C10	1.5169 (15)
O1—C15	1.2117 (14)	C10—C11	1.5233 (15)
O2—C15	1.3430 (14)	C10—C14	1.5241 (15)
O2—C16	1.4467 (14)	C10—H10A	0.9800
O3—C18	1.3440 (13)	C11—C12	1.3611 (15)
O3—C19	1.4519 (14)	C11—C18	1.4625 (15)
O4—C18	1.2233 (14)	C12—C22	1.4977 (16)
N1—N2	1.3556 (13)	C13—C14	1.3552 (15)
N1—C7	1.3628 (15)	C13—C21	1.5042 (16)
N1—H1N1	0.859 (18)	C14—C15	1.4707 (16)
N2—C8	1.3313 (15)	C16—C17	1.5060 (17)
N3—C12	1.3814 (15)	C16—H16A	0.9700
N3—C13	1.3888 (15)	C16—H16B	0.9700
N3—H1N3	0.909 (19)	C17—H17A	0.9600
C1—C2	1.3915 (16)	C17—H17B	0.9600
C1—C6	1.4021 (15)	C17—H17C	0.9600
C1—H1A	0.9300	C19—C20	1.5075 (18)
C2—C3	1.3819 (19)	C19—H19A	0.9700
C2—H2A	0.9300	C19—H19B	0.9700
C3—C4	1.3862 (17)	C20—H20A	0.9600
C4—C5	1.3940 (16)	C20—H20B	0.9600
C4—H4A	0.9300	C20—H20C	0.9600
C5—C6	1.3974 (17)	C21—H21A	0.9600
C5—H5A	0.9300	C21—H21B	0.9600
C6—C7	1.4670 (15)	C21—H21C	0.9600
C7—C9	1.3965 (14)	C22—H22A	0.9600
C8—C9	1.4108 (16)	C22—H22B	0.9600
C8—H8A	0.9300	C22—H22C	0.9600
			
C15—O2—C16	116.30 (9)	C14—C13—N3	118.15 (10)
C18—O3—C19	116.69 (9)	C14—C13—C21	128.07 (11)
N2—N1—C7	112.89 (9)	N3—C13—C21	113.78 (10)
N2—N1—H1N1	116.7 (12)	C13—C14—C15	123.77 (10)
C7—N1—H1N1	130.0 (12)	C13—C14—C10	119.60 (10)
C8—N2—N1	104.07 (9)	C15—C14—C10	116.55 (9)
C12—N3—C13	121.59 (9)	O1—C15—O2	122.63 (11)
C12—N3—H1N3	118.7 (11)	O1—C15—C14	124.73 (11)
C13—N3—H1N3	117.8 (11)	O2—C15—C14	112.54 (10)
C2—C1—C6	120.24 (11)	O2—C16—C17	105.52 (10)
C2—C1—H1A	119.9	O2—C16—H16A	110.6
C6—C1—H1A	119.9	C17—C16—H16A	110.6
C3—C2—C1	119.54 (11)	O2—C16—H16B	110.6
C3—C2—H2A	120.2	C17—C16—H16B	110.6
C1—C2—H2A	120.2	H16A—C16—H16B	108.8
C2—C3—C4	121.43 (11)	C16—C17—H17A	109.5
C2—C3—Cl1	119.55 (9)	C16—C17—H17B	109.5
C4—C3—Cl1	119.01 (10)	H17A—C17—H17B	109.5
C3—C4—C5	118.96 (12)	C16—C17—H17C	109.5
C3—C4—H4A	120.5	H17A—C17—H17C	109.5
C5—C4—H4A	120.5	H17B—C17—H17C	109.5
C4—C5—C6	120.71 (11)	O4—C18—O3	122.26 (10)
C4—C5—H5A	119.6	O4—C18—C11	126.36 (10)
C6—C5—H5A	119.6	O3—C18—C11	111.38 (9)
C5—C6—C1	119.10 (10)	O3—C19—C20	106.57 (10)
C5—C6—C7	120.83 (10)	O3—C19—H19A	110.4
C1—C6—C7	120.03 (10)	C20—C19—H19A	110.4
N1—C7—C9	106.36 (9)	O3—C19—H19B	110.4
N1—C7—C6	120.39 (10)	C20—C19—H19B	110.4
C9—C7—C6	133.25 (10)	H19A—C19—H19B	108.6
N2—C8—C9	112.86 (10)	C19—C20—H20A	109.5
N2—C8—H8A	123.6	C19—C20—H20B	109.5
C9—C8—H8A	123.6	H20A—C20—H20B	109.5
C7—C9—C8	103.82 (10)	C19—C20—H20C	109.5
C7—C9—C10	131.02 (10)	H20A—C20—H20C	109.5
C8—C9—C10	124.80 (10)	H20B—C20—H20C	109.5
C9—C10—C11	109.34 (9)	C13—C21—H21A	109.5
C9—C10—C14	113.54 (9)	C13—C21—H21B	109.5
C11—C10—C14	107.27 (8)	H21A—C21—H21B	109.5
C9—C10—H10A	108.9	C13—C21—H21C	109.5
C11—C10—H10A	108.9	H21A—C21—H21C	109.5
C14—C10—H10A	108.9	H21B—C21—H21C	109.5
C12—C11—C18	121.40 (10)	C12—C22—H22A	109.5
C12—C11—C10	118.82 (10)	C12—C22—H22B	109.5
C18—C11—C10	119.51 (9)	H22A—C22—H22B	109.5
C11—C12—N3	118.42 (10)	C12—C22—H22C	109.5
C11—C12—C22	127.72 (10)	H22A—C22—H22C	109.5
N3—C12—C22	113.79 (9)	H22B—C22—H22C	109.5
			
C7—N1—N2—C8	0.62 (13)	C14—C10—C11—C18	146.53 (10)
C6—C1—C2—C3	−0.41 (19)	C18—C11—C12—N3	−172.34 (10)
C1—C2—C3—C4	−0.9 (2)	C10—C11—C12—N3	13.70 (15)
C1—C2—C3—Cl1	178.92 (10)	C18—C11—C12—C22	10.73 (18)
C2—C3—C4—C5	1.7 (2)	C10—C11—C12—C22	−163.24 (11)
Cl1—C3—C4—C5	−178.15 (10)	C13—N3—C12—C11	19.94 (16)
C3—C4—C5—C6	−1.14 (19)	C13—N3—C12—C22	−162.71 (10)
C4—C5—C6—C1	−0.12 (18)	C12—N3—C13—C14	−22.38 (16)
C4—C5—C6—C7	177.48 (11)	C12—N3—C13—C21	157.22 (10)
C2—C1—C6—C5	0.91 (18)	N3—C13—C14—C15	167.42 (10)
C2—C1—C6—C7	−176.72 (11)	C21—C13—C14—C15	−12.11 (19)
N2—N1—C7—C9	−0.39 (13)	N3—C13—C14—C10	−9.08 (16)
N2—N1—C7—C6	178.92 (10)	C21—C13—C14—C10	171.38 (11)
C5—C6—C7—N1	−145.13 (11)	C9—C10—C14—C13	−83.80 (13)
C1—C6—C7—N1	32.45 (16)	C11—C10—C14—C13	37.11 (14)
C5—C6—C7—C9	33.95 (19)	C9—C10—C14—C15	99.45 (12)
C1—C6—C7—C9	−148.47 (13)	C11—C10—C14—C15	−139.64 (10)
N1—N2—C8—C9	−0.62 (13)	C16—O2—C15—O1	3.64 (18)
N1—C7—C9—C8	0.00 (12)	C16—O2—C15—C14	−172.93 (10)
C6—C7—C9—C8	−179.18 (12)	C13—C14—C15—O1	162.09 (13)
N1—C7—C9—C10	−173.26 (11)	C10—C14—C15—O1	−21.31 (17)
C6—C7—C9—C10	7.6 (2)	C13—C14—C15—O2	−21.42 (16)
N2—C8—C9—C7	0.40 (13)	C10—C14—C15—O2	155.18 (10)
N2—C8—C9—C10	174.21 (10)	C15—O2—C16—C17	176.01 (10)
C7—C9—C10—C11	116.66 (13)	C19—O3—C18—O4	−1.74 (17)
C8—C9—C10—C11	−55.36 (14)	C19—O3—C18—C11	178.61 (10)
C7—C9—C10—C14	−123.60 (12)	C12—C11—C18—O4	20.74 (18)
C8—C9—C10—C14	64.38 (14)	C10—C11—C18—O4	−165.33 (11)
C9—C10—C11—C12	84.14 (12)	C12—C11—C18—O3	−159.62 (10)
C14—C10—C11—C12	−39.39 (13)	C10—C11—C18—O3	14.30 (14)
C9—C10—C11—C18	−89.94 (12)	C18—O3—C19—C20	−178.84 (11)

### Hydrogen-bond geometry (Å, º)

*Cg*1 is the centroid of the N1/N2/C7–C9 ring.

**Table:**

*D*—H···*A*	*D*—H	H···*A*	*D*···*A*	*D*—H···*A*
N1—H1*N*1···O4^i^	0.857 (17)	2.078 (17)	2.9291 (14)	172.2 (17)
N3—H1*N*3···N2^ii^	0.908 (19)	2.184 (19)	3.0427 (14)	157.5 (15)
C5—H5*A*···O1	0.93	2.27	3.1988 (16)	177
C8—H8*A*···N3	0.93	2.61	3.2546 (15)	127
C22—H22*B*···N2^iii^	0.96	2.50	3.3741 (16)	151
C19—H19*B*···*Cg*1^iv^	0.96	2.79	3.5562 (14)	137

Symmetry codes: (i) *x*+1, *y*, *z*; (ii) −*x*, −*y*+2, −*z*; (iii) *x*−1, *y*, *z*; (iv) −*x*, −*y*+2, −*z*+1.
